# Supplementary material for: A genome-centric view of the role of the Acropora kenti microbiome in coral health and resilience
Source: Nat Commun. 2024 Apr 4;15:2902. doi: 10.1038/s41467-024-46905-5 (PMC10995205; doi:10.1038/s41467-024-46905-5)
Supplement: Supplementary file 1 — Supplementary Information [file 41467_2024_46905_MOESM1_ESM.pdf]

A genome-centric view of the role of the *Acropora kenti* microbiome in coral health and resilience

Supplementary Information

Lauren F. Messer[1,2]*, David G. Bourne[3,4], Steven J. Robbins[5], Megan Clay[1], Sara C. Bell[3,4], Simon J. McIlroy[1], Gene W. Tyson[1]*

1 Centre for Microbiome Research, School of Biomedical Sciences, Translational Research Institute, Queensland University of Technology, Brisbane, QLD, 4000, Australia

2 Division of Biological and Environmental Sciences, Faculty of Natural Sciences, University of Stirling, Stirling, FK9 4LA, Scotland

3 College of Science and Engineering, James Cook University, Townsville, QLD, 4810, Australia

4 Australian Institute of Marine Science, Townsville, QLD, 4810, Australia

5 Australian Centre for Ecogenomics, School of Chemistry and Molecular Biosciences, The University of Queensland, Brisbane, QLD, 4072, Australia

*Corresponding authors: Dr Lauren Messer; Email: lauren.messer@stir.ac.uk; Phone: +44 1786 467810; Professor Gene Tyson; Email: gene.tyson@qut.edu.au; Phone: +61 7 3443 7284

<u>This file includes:</u>

Supplementary Methods

Supplementary Note 1

Supplementary Note 2

Supplementary Note 3

Supplementary Note 4

Supplementary Note 5

Supplementary Fig. 1

Supplementary Fig. 2

Supplementary Table S1

Supplementary Table S2

Supplementary Methods

Quality control, coral host and Symbiodiniaceae contamination removal

To improve metagenomic assembly and the binning of microbial reads into metagenome-assembled genomes from *A. kenti* samples, reads mapping to reference genomes of the coral host and Symbiodiniaceae symbionts, and those representing PCR duplicates, were removed prior to processing. Briefly, adaptors were removed using Seqpurge (ngs-bits/2018_11) with the flags –ncut 0 and –qcut 0, and trimmed reads mapping to *A. kenti* [1] (http://aten.reefgenomics.org/), *Cladocopium* C15 sp. [2] (http://plut.reefgenomics.org/cladocopium_download/), and *Cladocopium goreaui* [3] (https://doi.org/10.14264/uql.2019.745), using CoverM (v0.2.0-alpha7; https://github.com/wwood/CoverM) with the flags --min-read-aligned-percent 0.75, --min-read-percent-identity 0.95 and --inverse used to retain all reads not meeting these criteria (i.e. those that are likely derived from the microbiome). To improve processing times, the host and Symbiodiniaceae removed reads were assembled using megahit (v1.1.4) [4] with the flag --min-contig-len 250 and read mapping alignments (BAM files) were generated using CoverM (v0.2.0-alpha7). The samtools Markdup workflow (http://www.htslib.org/doc/samtools-markdup.html) was followed to identify and remove PCR duplicates from the sorted BAM files after the removal of supplementary alignments (samtools flag -F2304; [5]). Finally, PCR duplicate-free BAM files were re-sorted using samtools sort -n and converted to fastq files using samtools fastq, then compressed using GNU gzip. GNU parallel was used throughout all analyses to speed up processing [6]. Metagenomic sequencing of microbiome enriched *A. kenti* holobiont samples from six reef sites (n = 22) resulted in a total of 621 Gb data. Between 56-90% of *A. kenti* metagenomic reads were removed through the QC process, giving rise to 118 Gb of sequence data for binning (Supplementary Fig. 1).

Evaluation of the negative control sample

Biological samples with high levels of host genome contamination and low microbial biomass can display decreased sensitivity in detecting rare microbial species [7], and consequently can be prone to the effects of contamination [8]. Sequencing of a DNA extraction and library preparation negative control enabled comparison between biological samples and contaminants. The 16S rRNA gene-based taxonomic profiles of the metagenomes were determined using GraftM [9] with the SILVA database, and for single copy ribosomal proteins using SingleM

(https://github.com/wwood/singlem), based on the Genome Taxonomy Database (GTDB) [10]. This analysis revealed discrete profiles between the samples and negative control, with ~83% of sequences in the negative control from the Enterobacteriaceae family, including the specific genera *Enterobacter* sp. (7.6%), *Escherichia* sp. (5.7%), *Klebsiella* sp. (12.4%), *Serratia* sp. (2.5%). In contrast, Enterobacteriaceae represented between 0.9-4.7% of sequences in the *A. kenti* samples, and 0.3-0.6% in the seawater samples. Mean relative abundances of specific genera were 0.2% and 0.0% for *Enterobacter* sp., 0.3% and 0.0% *Escherichia* sp., 0.0% and 0.1% *Klebsiella* sp., and 0.2% and 0.0% *Serratia* sp., in *A. kenti* and seawater samples respectively.

Reads from the control sample were mapped to the dereplicated MAGs using CoverM (v0.4.0) and the flags -m mean --min-read-percent-identity 0.5, --min-read-aligned-percent 0.5, representing lower stringency criteria than the *A. kenti* and seawater samples, to further confirm the validity of the MAGs. This demonstrated that 0% of reads from the control sample mapped to the MAGs, suggesting that none were introduced as contaminants during laboratory processing. However, three of the *A. kenti*-specific MAGs with normalised relative abundances between 0.03 - 5.5%, were classified as *Cutibacterium acnes* (Actinobacteriota; Fitzroy_MAG20, Russell_MAG30, and Magnetic_MAG20; formerly of the genus *Propionibacterium*) by GTDB, a common skin bacterium that has been observed in 16S rRNA sequencing studies from at least 15 coral species including *Acropora tenuis* [11], and recognised as a contaminant in low microbial biomass coral microbiome studies [12]. These MAGs do not appear to have been introduced during the laboratory processing, DNA extraction, or sequencing, as they were not detected in the control sample, thus they have not been removed from downstream analyses. Nevertheless, through the holobiont metabolic reconstruction and functional gene enrichment analyses conducted herein, they were not identified as playing any specific or key roles in holobiont community function. Ultimately, any supposition of their role in the *A. kenti* holobiont must be viewed with caution without validation of their physical positioning within the coral host [13].

Generation of the 'overall' dereplicated dataset and statistical analyses

The statistical tests carried out on the MAGs were driven by the following *a priori* hypotheses, i) the *A. kenti* microbiome would be taxonomically and functionally distinct to that of the surrounding seawater, ii) the composition of the *A. kenti* microbiome would demonstrate spatial heterogeneity due to latitudinal and/or local water quality gradients, and iii) *A. kenti*

microbiome function would vary due to latitudinal and/or local water quality differences. It is assumed that individual colonies from each sampling location represent individual biological replicates. Read mapping to closely related microbial strains can introduce cross-mapping of reads when calculating coverage and relative abundance, and therefore perform statistical analyses to test the specific hypotheses. Thus, an 'overall' dereplication step was performed on the 201 MAGs using dRep with default settings, resulting in 63 *A. kenti*-specific and 49 seawater-specific MAGs for biogeography analyses.

Supplementary Results and Discussion

Supplementary Note 1: Recovery of seawater MAGs

In total, 182 MAGs with quality scores ≥ 50 were recovered from the adjacent seawater samples (n = 6), resulting in 99 MAGs (86.7 ± 7.25 % mean completeness, and 2.89 ± 2.31 % mean contamination) after dereplication and quality filtering. These dereplicated MAGs were representative of > 30% of the genus-level representatives within the seawater microbiomes and spanned 7 bacterial and 1 archaeal phyla, including, Actinobacteriota (n = 18), Bacteroidota (n = 23), Cyanobacteriota (n = 11), Marinisomatota (n = 6), Planctomycetota (n = 2), Pseudomonadota (n = 27), candidate phylum SAR324 (n = 3), and Thermoplasmatota (n = 9), respectively. In contrast to the *A. kenti* samples, MAGs from the Cyanobacteriota comprised the most abundant lineages across the seawater samples. However, dominant seawater-associated lineages, such as the Pelagibacteraceae, were not represented within the recovered MAGs, in-line with previous genome-resolved metagenomic studies that have included coral reef seawater [2,14,15]. This is perhaps owing to the genomic complexity [16] and high alpha-diversity of microbial communities within seawater samples [17] which often precludes their assembly into contiguous sequences for binning.

Supplementary Note 2: *A. kenti* and seawater MAG specificity

Further investigation of the prevalence and relative abundance of MAGs across the dataset suggested that not all the *A. kenti*-derived MAGs were specific to the coral holobiont. In total, 20 of the *A. kenti*-derived MAGs were either prevalent (i.e. present in ≥ 50% of samples) within the seawater, displayed relative abundances in seawater ≥ 0.1 %, and/or displayed greater relative abundances within the seawater samples resulting in mean relative abundance ratios (*A. kenti*: seawater) of < 1. These 20 *A. kenti*-derived MAGs included representatives from the phyla Cyanobacteriota (16 MAGs from *Synechococcus_E* sp002724845, *Synechococcus C* sp.,

and *RCC307* sp012270465), Pseudomonadota (2 MAGs; *Luminiphilus* sp012270045 and *UBA8309* sp001627655), Planctomycetota (1 MAG; *UBA1268* sp002694955), and Bacteroidota (1 MAG; *UBA10066* sp003448535). Their recovery from *A. kenti* metagenomes at between 0.02 - 14.6% normalised relative abundances may indicate transient symbiotic relationships with the host, for example within coral mucus [18], or ingestion of these species by the host during times of heterotrophic feeding [19,20]. Due to their consistent prevalence, and higher relative abundances within the seawater samples, these MAGs may in fact represent carry-over of microorganisms from the surrounding seawater during sampling, or those ingested from seawater by the coral host. As one of the goals of the present study was to specifically determine functional roles of *A. kenti* microbial symbionts, the 20 *A. kenti* MAGs that were prevalent in seawater are considered within the seawater-specific dataset. The remaining 82 *A. kenti*-derived MAGs were considered specific to the *A. kenti* holobiont, however it must be noted that further evidence of the physical interaction between the taxa identified herein and the living tissues of *A. kenti* is required to confirm their positioning within the holobiont.

Supplementary Note 3: Molecular mechanisms indicative of a host-associated lifestyle within *A. kenti*-specific MAGs

Investigation of the specific genomic features of individual lineages hinted at their putative niches within the *A. kenti* holobiont. For example, the *A. kenti* MAGs with the smallest predicted genome sizes of 0.68 - 0.89 Mbp, and lowest numbers of predicted genes at 588 - 820, belonged to the intracellular Firmicutes family Mycoplasmataceae (3 MAGs with quality scores between 87 - 92%; Fitzroy_MAG11, Magnetic_MAG17, and Russell_MAG37). This lineage has previously been visualised within the tissues of cold-water corals [21], but they are uncommon within tropical scleractinian coral microbiome taxonomic surveys [11]. Their presence across three different reef sites at maximum relative abundances of 9.1% suggests they may also occur as important intracellular symbionts within *A. kenti*, but *in situ* visualisation necessary to confirm this hypothesis. In contrast, MAGs from the widely distributed invertebrate-associated genus *Endozoicomonas* sp. (9 MAGs with quality scores between 86 - 95%; Fitzroy_MAG7, Fitzroy_MAG17, Russell_MAG1, Russell_MAG12, Dunk_MAG4, Pelorus_MAG18, Pandora_MAG11, Pandora_MAG22, Magnetic_MAG22) displayed larger genome sizes of 3.75 - 5.84 Mbp and encoded between 3483 - 4549 predicted genes, perhaps reflecting the host-specific, yet flexible niche of Endozoicomonadaceae within

coral mucus and tissues [24], and a necessary extensive metabolic repertoire. These MAGs were prevalent across the six reef sites with maximum relative abundances of between 8.5 - 23%, supporting the notion that they are key symbionts within the *A. kenti* holobiont [23].

Supplementary Note 4: Nitrogen metabolism within the *A. kenti* microbiome

Many of the lineages identified as playing a role in holobiont nitrogen cycling displayed patchy distributions across the *A. kenti* samples, questioning their overall importance. However, some are putative facultative anaerobes suggesting their positioning is not within *A. kenti* tissues but rather they may reside within the coral skeleton [24]. The skeletal microbiome was not targeted in our microbial enrichment protocol, and thus they may be underrepresented in our dataset, though some carry-over may have occurred due to the harsh nature of the air-blasting. As an example, several *A. kenti*-specific MAGs encoding nitrogen fixation, including novel genera within the families Desulfovibrionaceae (Fitzroy_MAG12), Sedimenticolaceae (Pandora_MAG18), and Methyloligellaceae (Pelorus_MAG5), and the genus *Desulforhopalus* (Pandora_MAG12), were only present in the sample they were derived from at between 1.2 - 2.7% relative abundance. While 1 MAG of the well-known coral-associated species, *Chlorobium_A marina* (Russell_MAG40), was present in four samples, three of these were from Russell Island. Moreover, although 1 MAG from the genus *Desulfobacter sp.* (Magnetic_MAG13) was detected in five samples, relative abundances outside of the sample it was derived from were < 0.5%. These results contrast with previous surveys of coral-associated diazotrophs, in which Alphaproteobacteria typically dominate and appear to be widespread symbionts [25–28]. Rather, herein *Nitratireductor aquibiodomus* (Pandora_MAG31), displayed prevalence across the *A. kenti* samples. In previous research, *Nitratireductor aquibiodomus* has been associated with coral Black Band Disease [29], but here it was present in 60% of samples at relative abundances between 0.1-7.4%, suggesting it may play a key role in nitrogen cycling in healthy *A. kenti* colonies.

Supplementary Note 5: Genomic evidence of autotrophic carbon fixation in *A. kenti* MAGs

The reverse tricarboxylic acid cycle (rTCA) was encoded by 13 MAGs of five phyla, but the key enzyme ATP citrate lyase was only identified in two: *Chlorobium_A marina* (Russell_MAG40) and Nitrospirota *Bin75* sp. (Magnetic_MAG5). The modified hydroxypropionate-hydroxybutyrate cycle of *Nitrosopumilus maritimus* [30], was encoded by the

archaeal *JACEMX01 sp.* (Pandora_MAG19). While genes of the Calvin-Benson-Bassham (CBB) cycle were encoded by 10 MAGs from the Pseudomonadota (n = 9) and Bacteroidota (n = 1), including *Chlorobium_A marina* (Russell_MAG40) and *Nitratireductor aquibiodomus* (Pandora_MAG31). However, phylogenetic placement revealed that only two *A. kenti*-specific MAGs encoded the ribulose-bisphosphate carboxylase (RuBisCO) enzyme functional within the CBB cycle. Namely, *Chlorobium_A marina* (Russell_MAG40) and the Sedimenticolaceae MAG (Pandora_MAG18), which encodes two copies of the Form I RuBisCO subunit *rbcL* but has an incomplete CBB cycle (71% completeness).

Supplementary Figures

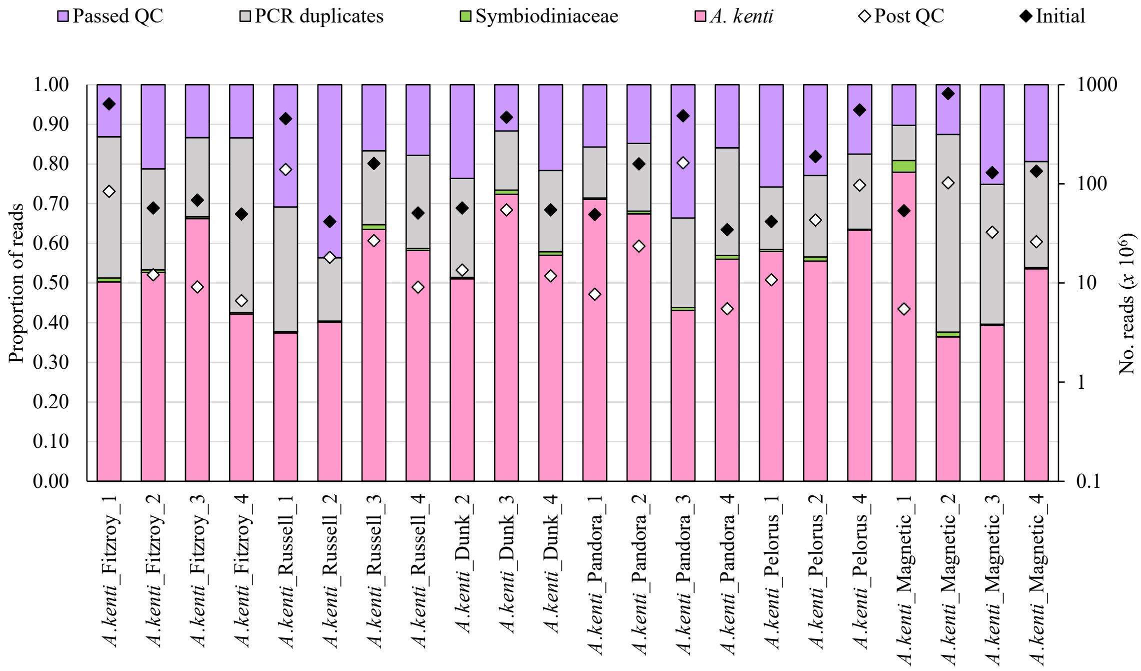

Supplementary Fig. 1. Results of the rigorous *A. kenti* metagenomics quality control workflow. Proportions of reads removed due to PCR duplicates and mapping to Symbiodiniaceae and *A. kenti* genomes, are shown for each *A. kenti* sample, in addition to the proportions of reads that were retained ('Passed QC') for MAG recovery. The total numbers of reads are shown for the initial metagenomes ('Initial') and those remaining after quality control ('Post QC'). Source data are provided as a Source Data file.

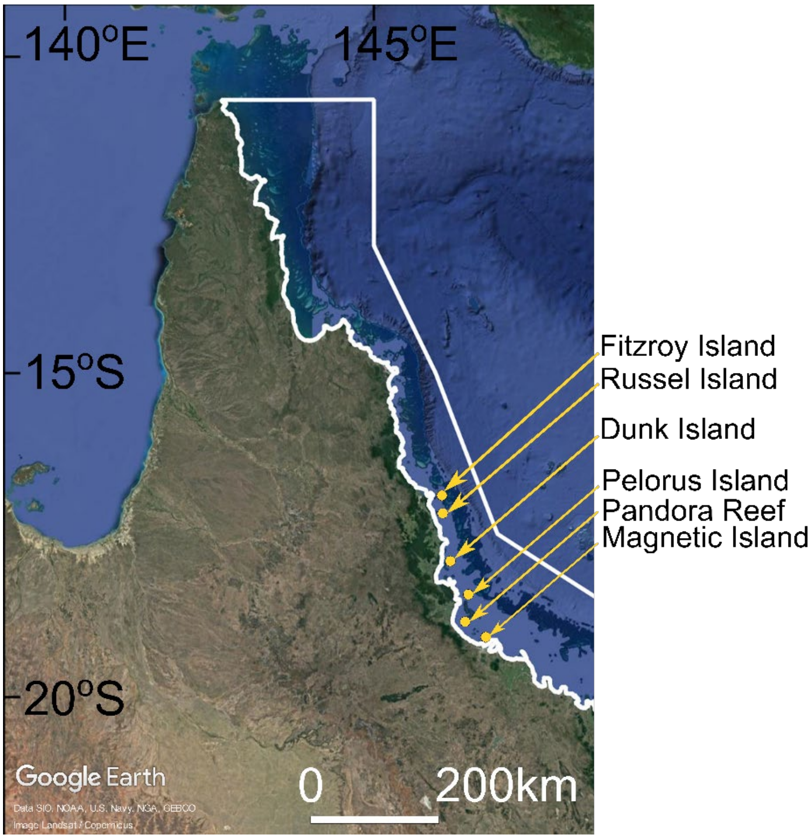

Supplementary Fig. 2. Map showing location of six *A. kenti* and adjacent seawater sampling sites of the inshore Great Barrier Reef. At each site, four *A. kenti* colonies and one sample of seawater was analysed for genome-resolved metagenomics. The satellite image was obtained from Google Earth and modified in Inkscape.

Supplementary Tables

Supplementary Table 1. Significant differences in microbial community composition. Post-hoc pairwise comparisons of *A. kenti* (n=63) and seawater (n=49) MAG-based community composition based on the Island site origin of the samples (n=28). Significant differences were identified using pairwise factor fitting to an ordination. P-values were corrected for multiple testing made by controlling for the False Discovery Rate.

**Pairwise Tests**	**F Model**	**R^2**	**P-adj.**
Fitzroy vs Dunk	11.182	0.691	0.053
Fitzroy vs Russell	2.740	0.313	0.053
Fitzroy vs Magnetic	3.931	0.396	0.053
Fitzroy vs Pandora	2.812	0.319	0.053
Fitzroy vs Pelorus	6.775	0.575	0.053
Fitzroy vs Seawater	121.490	0.938	**0.038**
Dunk vs Russell	4.918	0.496	0.053
Dunk vs Magnetic	6.977	0.583	0.053
Dunk vs Pandora	2.233	0.309	0.105
Dunk vs Pelorus	2.962	0.425	0.111
Dunk vs Seawater	79.884	0.919	**0.038**
Russell vs Magnetic	3.628	0.377	0.053
Russell vs Pandora	1.375	0.186	0.309
Russell vs Pelorus	2.378	0.322	0.073
Russell vs Seawater	81.875	0.911	**0.038**
Magnetic vs Pandora	2.320	0.279	0.053
Magnetic vs Pelorus	4.674	0.483	0.053
Magnetic vs Seawater	95.734	0.923	**0.038**
Pandora vs Pelorus	1.367	0.215	0.242
Pandora vs Seawater	59.416	0.881	**0.038**
Pelorus vs Seawater	74.534	0.914	0.053

Supplementary Table 2. Heterogeneity in the *A. kenti* microbiome underpinned by water quality. Post-hoc pairwise comparisons of *A. kenti* MAG-based (n=63) community composition based on the water quality categories of samples (n=22). Significant differences were identified using pairwise factor fitting to an ordination. P-values were corrected for multiple testing made by controlling for the False Discovery Rate.

**Pairwise Tests**	**F Model**	**R^2**	**P-adj.**
North Marine vs North Plume	4.5	0.334	**0.033**
North Marine vs Coastal	3.5	0.262	**0.020**
North Marine vs South Plume	2.2	0.177	0.061
North Marine vs South Marine	3.7	0.293	**0.030**
North Plume vs Coastal	5.2	0.512	0.050
North Plume vs South Plume	1.4	0.221	0.209
North Plume vs South Marine	2.3	0.361	0.125
Coastal vs South Plume	2.6	0.303	0.050
Coastal vs South Marine	4.7	0.486	0.050
South Plume vs South Marine	1.5	0.233	0.193

References

1. Cooke, I. *et al.* Genomic signatures in the coral holobiont reveal host adaptations driven by Holocene climate change and reef specific symbionts. *Sci Adv* **6**, (2020).

2. Robbins, S. J. *et al.* A genomic view of the reef-building coral Porites lutea and its microbial symbionts. *Nat Microbiol* **4**, 2090–2100 (2019).

3. Chen, Y., González-Pech, R. A., Stephens, T. G., Bhattacharya, D. & Chan, C. X. Evidence That Inconsistent Gene Prediction Can Mislead Analysis of Dinoflagellate Genomes. *J. Phycol.* **56**, 6–10 (2020).

4. Li, D., Liu, C.-M., Luo, R., Sadakane, K. & Lam, T.-W. MEGAHIT: an ultra-fast single-node solution for large and complex metagenomics assembly via succinct de Bruijn graph. *Bioinformatics* **31**, 1674–1676 (2015).

5. Li, H. *et al.* The Sequence Alignment/Map format and SAMtools. *Bioinformatics* **25**, 2078–2079 (2009).

6. Tange, O. GNU Parallel: The Command-Line Power Tool.

7. Pereira-Marques, J. *et al.* Impact of Host DNA and Sequencing Depth on the Taxonomic Resolution of Whole Metagenome Sequencing for Microbiome Analysis. *Front. Microbiol.* **10**, 1277 (2019).

8. Eisenhofer, R. *et al.* Contamination in Low Microbial Biomass Microbiome Studies: Issues and Recommendations. *Trends Microbiol.* **27**, 105–117 (2019).

9. Boyd, J. A., Woodcroft, B. J. & Tyson, G. W. GraftM: a tool for scalable, phylogenetically informed classification of genes within metagenomes. *Nucleic Acids Res.* **46**, e59 (2018).

10. Parks, D. H. *et al.* A standardized bacterial taxonomy based on genome phylogeny substantially revises the tree of life. *Nat. Biotechnol.* **36**, 996–1004 (2018).

11. Huggett, M. J. & Apprill, A. Coral microbiome database: Integration of sequences reveals high diversity and relatedness of coral-associated microbes. *Environ. Microbiol. Rep.* **11**, 372–385 (2019).

12. Kellogg, C. A. Microbiomes of stony and soft deep-sea corals share rare core bacteria. *Microbiome* **7**, 90 (2019).

13. D Ainsworth, T. *et al.* The coral core microbiome identifies rare bacterial taxa as ubiquitous endosymbionts. *ISME J.* **9**, 2261–2274 (2015).

14. Glasl, B. *et al.* Comparative genome-centric analysis reveals seasonal variation in the function of coral reef microbiomes. *ISME J.* **14**, 1435–1450 (2020).

15. Robbins, S. J. *et al.* A genomic view of the microbiome of coral reef demosponges. *ISME J.* (2021) doi:10.1038/s41396-020-00876-9.

16. Delmont, T. O. *et al.* Single-amino acid variants reveal evolutionary processes that shape the biogeography of a global SAR11 subclade. *Elife* **8**, (2019).

17. Pachiadaki, M. G. *et al.* Charting the Complexity of the Marine Microbiome through Single-Cell Genomics. *Cell* **179**, 1623-1635.e11 (2019).

18. Marchioro, G. M. *et al.* Microbiome dynamics in the tissue and mucus of acroporid corals differ in relation to host and environmental parameters. *PeerJ* **8**, e9644 (2020).

19. Meunier, V. *et al.* Diazotroph-Derived Nitrogen Assimilation Strategies Differ by Scleractinian Coral Species. *Frontiers in Marine Science* **8**, 1018 (2021).

20. Tremblay, P., Naumann, M. S., Sikorski, S., Grover, R. & Ferrier-Pagès, C. Experimental assessment of organic carbon fluxes in the scleractinian coral Stylophora pistillata during a thermal and photo stress event. *Mar. Ecol. Prog. Ser.* **453**, 63–77 (2012).

21. Neulinger, S. C. *et al.* Tissue-associated "Candidatus Mycoplasma corallicola" and filamentous bacteria on the cold-water coral Lophelia pertusa (Scleractinia). *Appl. Environ. Microbiol.* **75**, 1437–1444 (2009).

22. Pollock, F. J. *et al.* Coral-associated bacteria demonstrate phylosymbiosis and cophylogeny. *Nat. Commun.* **9**, 4921 (2018).

23. Glasl, B., Smith, C. E., Bourne, D. G. & Webster, N. S. Disentangling the effect of host-genotype and environment on the microbiome of the coral Acropora tenuis. *PeerJ* **7**, e6377 (2019).

24. Pernice, M. *et al.* Down to the bone: the role of overlooked endolithic microbiomes in reef coral health. *ISME J.* **14**, 325–334 (2020).

25. Lema, K. A., Bourne, D. G. & Willis, B. L. Onset and establishment of diazotrophs and other bacterial associates in the early life history stages of the coral Acropora millepora. *Mol. Ecol.* **23**, 4682–4695 (2014).

26. Glaze, T. D., Erler, D. V. & Siljanen, H. M. P. Microbially facilitated nitrogen cycling in tropical corals. *ISME J.* (2021) doi:10.1038/s41396-021-01038-1.

27. Lesser, M. P., Morrow, K. M., Pankey, S. M. & Noonan, S. H. C. Diazotroph diversity and nitrogen fixation in the coral Stylophora pistillata from the Great Barrier Reef. *ISME J.* **12**, 813–824 (2018).

28. Liang, J. *et al.* Diazotroph Diversity Associated With Scleractinian Corals and Its Relationships With Environmental Variables in the South China Sea. *Front. Physiol.* **11**, 615 (2020).

29. Henao, J. *et al.* Genome sequencing of three bacteria associated to black band disease from a Colombian reef-building coral. *Genom Data* **11**, 73–74 (2017).

30. Könneke, M. *et al.* Isolation of an autotrophic ammonia-oxidizing marine archaeon. *Nature* **437**, 543–546 (2005).
