# Supplementary material for: A genome-centric view of the role of the Acropora kenti microbiome in coral health and resilience
Source: Nat Commun. 2024 Apr 4;15:2902. doi: 10.1038/s41467-024-46905-5 (PMC10995205; doi:10.1038/s41467-024-46905-5)
Supplement: Supplementary file 3 — Description of Additional Supplementary Files [file 41467_2024_46905_MOESM3_ESM.pdf]

Description of Additional Supplementary Files

Title: Supplementary Data 1

Description: Taxonomic, quality, and genomic feature metadata for the 201 metagenome-assembled genomes described in this study.

Title: Supplementary Data 2

Description: Output from functional gene enrichment analysis between *A. kenti*-specific (n=82) and seawater-specific (n=119) metagenome-assembled genomes, based on a two-sided Fisher's Exact Test with correction for multiple testing controlled by False Discovery Rate analysis, as implemented by EnrichM 'enrichment'.

Title: Supplementary Data 3

Description: Identification of dimethylsulfoniopropionate metabolism genes in the 201 metagenome-assembled genomes described in this study. Black highlight indicates putatively functional genes identified using GraftM and confirmed using BLASTP against the NCBI database.

Title: Supplementary Data 4

Description: Output from functional gene clusters enrichment analysis between overall dereplicated *A. kenti*-specific metagenome-assembled genomes (n=63) across water quality categories (n=22 samples), as implemented by EnrichM 'enrichment' using a two-sided Mann Whitney U statistical test.

Title: Supplementary Data 5

Description: Model output from the constrained ordination of *A. kenti* (n=22 samples) and seawater (n=6 samples) community composition (n=112 metagenome-assembled genomes) based on the Island site origin of the samples, using the capscale function of vegan.

Title: Supplementary Data 6

Description: Model output from the 'goodness of fit' significance testing of the constrained ordination of *A. kenti* (n=22 samples) and seawater (n=6 samples) community composition (n=112 metagenome-assembled genomes), based on the Island site origin factor using the envfit function of vegan.

Title: Supplementary Data 7

Description: Model output from the constrained ordination of *A. kenti* (n=22 samples) community composition (n=63 metagenome-assembled genomes), based on the water quality category factor using the capscale function of vegan.

Title: Supplementary Data 8

Description: Model output from the 'goodness of fit' significance testing of the constrained ordination of *A. kenti* (n=22 samples) community composition (n=63 metagenome-assembled genomes), based on the water quality category factor using the envfit function of vegan.
