# Supplementary material for: A genome-centric view of the role of the Acropora kenti microbiome in coral health and resilience
Source: Nat Commun. 2024 Apr 4;15:2902. doi: 10.1038/s41467-024-46905-5 (PMC10995205; doi:10.1038/s41467-024-46905-5)
Supplement: Supplementary file 9 — Reporting Summary [file 41467_2024_46905_MOESM9_ESM.pdf]

nature portfolio

Corresponding author(s): Dr Lauren Messer; Prof. Gene Tyson

Last updated by author(s): 20/02/2024

Reporting Summary

Nature Portfolio wishes to improve the reproducibility of the work that we publish. This form provides structure for consistency and transparency in reporting. For further information on Nature Portfolio policies, see our Editorial Policies and the Editorial Policy Checklist.

Statistics

For all statistical analyses, confirm that the following items are present in the figure legend, table legend, main text, or Methods section.

n/a	Confirmed	
☐	☒	The exact sample size (*n*) for each experimental group/condition, given as a discrete number and unit of measurement
☐	☒	A statement on whether measurements were taken from distinct samples or whether the same sample was measured repeatedly
☐	☒	The statistical test(s) used AND whether they are one- or two-sided<br>*Only common tests should be described solely by name; describe more complex techniques in the Methods section.*
☐	☒	A description of all covariates tested
☐	☒	A description of any assumptions or corrections, such as tests of normality and adjustment for multiple comparisons
☐	☒	A full description of the statistical parameters including central tendency (e.g. means) or other basic estimates (e.g. regression coefficient) AND variation (e.g. standard deviation) or associated estimates of uncertainty (e.g. confidence intervals)
☐	☒	For null hypothesis testing, the test statistic (e.g. *F*, *t*, *r*) with confidence intervals, effect sizes, degrees of freedom and *P* value noted<br>*Give P values as exact values whenever suitable.*
☒	☐	For Bayesian analysis, information on the choice of priors and Markov chain Monte Carlo settings
☒	☐	For hierarchical and complex designs, identification of the appropriate level for tests and full reporting of outcomes
☐	☒	Estimates of effect sizes (e.g. Cohen's *d*, Pearson's *r*), indicating how they were calculated

Our web collection on statistics for biologists contains articles on many of the points above.

Software and code

Policy information about availability of computer code

Data collection	No code was used to collect data used in this study.
Data analysis	All code used in the analysis detailed in the Methods and Supplementary Methods is publicly available, either through the original cited publication or through links provided in the text to the respective GitHub pages. The metagenomic analysis detailed in the manuscript is briefly described here, and included the following: adaptors were removed using Seqpurge (ngs-bits/2018_11), and trimmed reads were mapped to the host and Symbiodiniaceae genomes using CoverM (v0.2.0-alpha7; https://github.com/wwood/CoverM). Microbial reads were assembled using megahit (v1.1.4) and read mapping alignments (BAM files) were generated using CoverM (v0.2.0-alpha7). The samtools Markdup workflow (http://www.htslib.org/doc/samtools-markdup.html) was used to remove PCR duplicates. The final 'clean' microbial reads were assembled using the metaspades.py script of Spades (v3.13.0). Quality controlled reads were mapped to the resulting scaffolds using CoverM (v0.2.0-alpha6; https://github.com/wwood/CoverM), and the generated BAM files and scaffolds were used as input to the custom ensemble binning tool UniteM (v1.0.0; https://github.com/dparks1134/UniteM/), which produced a non-redundant set of MAGs from the binning methods: GroopM2 (https://github.com/Ecogenomics/GroopM), MaxBin, MetaBAT2 and MetaBAT1. Contigs identified as outliers based on GC content and tetranucleotide distance were removed from the MAGs using RefineM (v0.0.24; https://github.com/dparks1134/RefineM). The quality (defined as completeness - 3 × contamination) of the resulting MAGs was determined using CheckM (v1.1.2). MAGs were assigned taxonomy based on the standardised taxonomic ranks of the Genome Taxonomy Database (GTDB, r202) using GTDB-tk (v1.5.0). To reduce redundancy between biological replicates, but not to eliminate beta-diversity, MAGs with a quality score ≥ 50 were dereplicated based on the sampling site they were recovered from using dRep (v2.5.4), including the additional quality filtering step of only retaining MAGs ≥ 75% complete. The extent of the microbial communities encompassed by the dereplicated MAGs was determined using SingleM 'appraise' (v0.12.1; https://github.com/wwood/singlem). EnrichM (v0.4.15; https://github.com/geronimp/enrichM) was used to annotate predicted proteins of all MAGs, host and Symbiodiniaceae, with carbohydrate active enzymes (CAZy), Kyoto Encyclopedia of Genes and Genomes (KEGG) Orthology (KOs), and protein families (PFams). To identify functional motifs in specific genes of interest (dddX, dmdA,

cuMMO, nxrAB, nifH, rbcL, aclA), GraftM (v0.13.1; https://data.ace.uq.edu.au/public/graftm/7/) was used to search the predicted proteins of MAGs and place then into a phylogenetic tree, using publicly available GraftM packages (found at the link mentioned previously).

For manuscripts utilizing custom algorithms or software that are central to the research but not yet described in published literature, software must be made available to editors and reviewers. We strongly encourage code deposition in a community repository (e.g. GitHub). See the Nature Portfolio guidelines for submitting code & software for further information.

Data

Policy information about availability of data

All manuscripts must include a data availability statement. This statement should provide the following information, where applicable:

- Accession codes, unique identifiers, or web links for publicly available datasets
- A description of any restrictions on data availability
- For clinical datasets or third party data, please ensure that the statement adheres to our policy

The sequence data generated in this study have been submitted to NCBI under BioProject PRJNA545004 (https://www.ncbi.nlm.nih.gov/bioproject/?term=PRJNA545004),with BioSample accessions spanning SAMN37503415-SAMN37503442 (e.g. https://www.ncbi.nlm.nih.gov/biosample/ SAMN37503415). Environmental water quality data obtained according to the Great Barrier Reef Marine Monitoring Program are publicly available at https://www.aims.gov.au/data. The A. kenti genome used in this study is available at http://aten.reefgenomics.org/, Cladocopium C15 sp. at http://plut.reefgenomics.org/cladocopium_download/, and Cladocopium goreaui https://doi.org/10.14264/uql.2019.745. The A. kenti and seawater MAGs metadata (i.e. taxonomy, quality, genomic features) generated in this study are provided in the Supplementary Data files. The Genome Taxonomy Database is publicly available at https://gtdb.ecogenomic.org/downloads. GraftM packages used in this study are available at https://data.ace.uq.edu.au/public/graftm/7/. Source data are provided with this paper.

Research involving human participants, their data, or biological material

Policy information about studies with human participants or human data. See also policy information about sex, gender (identity/presentation), and sexual orientation and race, ethnicity and racism.

Reporting on sex and gender	N/A
Reporting on race, ethnicity, or other socially relevant groupings	N/A
Population characteristics	N/A
Recruitment	N/A
Ethics oversight	N/A

Note that full information on the approval of the study protocol must also be provided in the manuscript.

Field-specific reporting

Please select the one below that is the best fit for your research. If you are not sure, read the appropriate sections before making your selection.

☐ Life sciences ☐ Behavioural & social sciences ☒ Ecological, evolutionary & environmental sciences

For a reference copy of the document with all sections, see nature.com/documents/nr-reporting-summary-flat.pdf

Ecological, evolutionary & environmental sciences study design

All studies must disclose on these points even when the disclosure is negative.

Study description	The branching coral Acropora kenti (n=24) and adjacent seawater (n=6), were sampled from six inshore reef sites situated along two gradients of increasing water quality, during a voyage of the RV Cape Ferguson to the Great Barrier Reef. A. kenti samples were processed to enrich the microbial component of the coral holobiont for metagenomic analysis (n = 22; 2 samples failed to produce sufficient DNA, see 'Data exclusions') to elucidate the functional roles of the microbiome. Samples collected from the adjacent seawater (n=6) were used to generate free-living 'control' metagenomes. Holobiont-wide metabolic reconstruction was used to determine the overall functional contributions of the associated microorganisms and metagenome-assembled genomes were classified as either A. kenti-specific (n=82) or seawater-specific (n=119) for statistical comparisons of genomic features and functional gene content. The northern sampling gradient, spanning poor to improved water quality, included Dunk Island (n=4 A. kenti; n=1 seawater), Russell Island (n=4; n=1), and Fitzroy Island (n=4; n=1), respectively. The southern sampling gradient encompassed the sites Magnetic Island (n=4; n=1), Pandora Reef (n=4; n=1), and Pelorus Island (n=4; n=1), from poor to improved water quality. These sites were classified as marine (Fitzroy, Russell, and Pelorus Islands), river-plume impacted (Dunk Island and Pandora Reef), and coastal (Magnetic Island) factors for statistical comparisons. Biogeography analyses were employed to determine the effects of water quality and latitude on A. kenti microbiome composition and function.
Research sample	A. kenti is widely distributed across the Great Barrier Reef and represents an environmentally sensitive coral species, negatively impacted by nutrient enrichment and thermal stress. This species has been the subject of active reef restoration initiatives, host genome and Symbiodiniaceae sequencing (datasets available at http://aten.reefgenomics.org/ and https://doi.org/10.14264/uql.2019.745, respectively), and previous studies exploring the taxonomic composition of the microbiome. A. kenti is emerging as an

	ideal coral species to further refine models for coral-host-microbiome symbioses. Adult colonies of A. kenti > 5 years (identified based on their size, morphology, and an age of first reproduction of 3 years) were chosen as the focus of this study. A. kenti were sampled directly from the environment and were not manipulated prior to microbiome enrichment.
Sampling strategy	At each site, branches from four adult A. kenti colonies were fragmented (~10 × 10 cm2) by trained coral biologists using SCUBA, employing a hammer and chisel to detach the fragment from the colony. No sample size calculation was performed prior to sampling, previous knowledge and experience of coral-associated microbial community variability informed the selection of four colonies per sampling site, while limiting the impact of our sampling on the individual reef ecosystems. The colonies were brought on-board the research vessel for input into a microbial enrichment protocol. One sample (5 L) from the surrounding seawater was collected from each site and 5 µm pre-filtered onto a 0.2 µm Sterivex filter using a peristaltic pump. A. kenti tissues were removed from the skeleton using a sterile air pick in calcium and magnesium-free artificial seawater and the blastate was homogenised using a sterile glass dounce. Two enzyme treatments were applied to break down coral mucus and tissue structures, prior to sequential filtration and slow speed centrifugation to remove intact tissues, skeletal debris, and Symbiodiniaceae cells. Additional filtrations through 8.0 µm and 5 µm membrane filters were used to produce a 'microbiome' enriched cell pellet for DNA extraction.
Data collection	Samples were collected by Sara Bell and David Bourne, with assistance from several researchers detailed in the Acknowledgements including, Johnston Davidson, Paul Costello, Russell Carpenter, and Kathy Morrow. Sara Bell and David Bourne maintained laboratory notebooks and converted these to digital records, detailing the sample collection data and methodology, and the steps of the microbiome enrichment protocol. Lauren Messer and Steven Robbins also maintained laboratory notebooks and digital records of the DNA extraction, clean-up, and library preparation procedures. Original copies of laboratory notebooks are held at the respective research institutions.
Timing and spatial scale	The two water quality sampling gradients collectively spanned 2° of latitude and 220km. Samples were collected from randomly selected A. kenti colonies from the respective sites during the tropical wet season, between 20th - 23rd February 2015 (permit number G14/36802.1) following the route and methodology detailed by Cooke and colleagues (Cooke, et al. Genomic signatures in the coral holobiont reveal host adaptations driven by Holocene climate change and reef specific symbionts. Sci Adv 6, (2020)). Due to the logistics of sampling six different coral reef habitats from an oceanographic research vessel, samples were collected once from different locations over several days including Fitzroy Island, 20 February 2015; Russell Island, 21 February 2015, Dunk Island, 21 February 2015; Pelorus Island, 22 February 2015, Pandora Reef, 22 February 2015; and Magnetic Island, 23 February 2015.
Data exclusions	No data were excluded, however two A. kenti samples (replicate 1 from Dunk Island and replicate 3 from Pelorus Island) and 1 DNA extraction and library preparation negative control did not produce sufficient DNA following library preparation, and therefore these samples were not sequenced.
Reproducibility	The methods used to collect the samples, produce the extracted DNA and metagenomic libraries, are established protocols that have been i) optimised over several years and ii) performed multiple times on corals by the research team and highly trained laboratory technicians. They are proven to be highly reproducible across different research groups and laboratories world-wide for microbial community composition analyses. The final workflow used to analyse the metagenomic data is also highly reproducible as it employed publicly available software and code and comprises standard methods used widely throughout the field of metagenomics.
Randomization	Samples were collected from randomly selected A. kenti colonies from the respective sites following the route and methodology detailed by Cooke and colleagues (Cooke, et al. Genomic signatures in the coral holobiont reveal host adaptations driven by Holocene climate change and reef specific symbionts. Sci Adv 6, (2020)). A. kenti samples were organised into experimental groups based on their sampling site of origin and the classifications of water quality at these sites (i.e. marine = Fitzroy, Russell, and Pelorus Islands; river-plume impacted = Dunk Island and Pandora Reef; and coastal = Magnetic Island). A. kenti and seawater-specific metagenome assembled genomes were classified following three a posteriori criteria: an A. kenti MAG should have a greater mean relative abundance in A. kenti compared to seawater samples (A. kenti: seawater relative abundance ratio > 1), should display < 50% prevalence within the seawater, and represent < 0.1% relative abundance in each seawater sample.
Blinding	Blinding was not required in this study. A. kenti colonies needed to be identified to relate the microbiome to the environmental metadata and control seawater metagenomes.

Did the study involve field work? ☒ Yes ☐ No

Field work, collection and transport

Field conditions	Reef conditions were consistent with the sampling season and are captured by the environmental water quality data obtained according to the Great Barrier Reef Marine Monitoring Program using standard protocols, publicly available at https://www.aims.gov.au/data.
Location	Inshore GBR reef sites included Fitzroy Island (-16.92315, 145.99625), Russell Island (-17.2269667, 146.0903667), Dunk Island (-17.9262833, 146.1461333), Pelorus Island (-18.5405833, 146.48855), Pandora Reef (-18.8169601, 146.4385087), and Magnetic Island (-19.15495, 146.8684833).
Access & import/export	All sample collections were performed in accordance with the Great Barrier Reef Marine Park Authority (GBRMPA) regulations, under permit number G14/36802.1.
Disturbance	The size of the colony fragments collected were in-line with the sampling recommendations from GBRMPA, and these have been shown to have minimal disturbance to coral populations. As colonial organisms, the colony heals and regrows these regions overtime, with branching corals such as A. kenti representing a relatively fast growing species. Collectively this limited the disturbance on the natural reef ecosystem.

Reporting for specific materials, systems and methods

We require information from authors about some types of materials, experimental systems and methods used in many studies. Here, indicate whether each material, system or method listed is relevant to your study. If you are not sure if a list item applies to your research, read the appropriate section before selecting a response.

Materials & experimental systems

n/a	Involved in the study
☒	☐ Antibodies
☒	☐ Eukaryotic cell lines
☒	☐ Palaeontology and archaeology
☐	☒ Animals and other organisms
☒	☐ Clinical data
☒	☐ Dual use research of concern
☒	☐ Plants

Methods

n/a	Involved in the study
☒	☐ ChIP-seq
☒	☐ Flow cytometry
☒	☐ MRI-based neuroimaging

Animals and other research organisms

Policy information about studies involving animals; ARRIVE guidelines recommended for reporting animal research, and Sex and Gender in Research

Laboratory animals	The study did not involve laboratory animals.
Wild animals	Adult colonies of A. kenti > 5 years (identified based on their size, morphology, and an age of first reproduction of 3 years) were sampled from the field for this study. Colonies were fragmented (~10 × 10 cm2) underwater using hammer and chisel by trained coral biologists using SCUBA, leaving the majority of the adult colony intact and undistrubed. A. kenti colony fragments were transported from the reef to the research vessel in plastic baskets, being retained underwater before transferring to the research vessel laboratory for processing.
Reporting on sex	Acroporid corals are broadcast spawners, meaning each colony is a hermaphrodite. Sex-based analyses are therefore not relevant to this study.
Field-collected samples	Samples collected from the field were processed immediately and were not maintained in the laboratory.
Ethics oversight	The Great Barrier Reef Marine Park Authority (GBRMPA), Australian Institute of Marine Science, and University of Queensland collectively approved and provided guidance on the study protocol.

Note that full information on the approval of the study protocol must also be provided in the manuscript.
